# Super-Resolution Microscopy Reveals a Nanoscale Organization of Acetylcholine Receptors for Trans-Synaptic Alignment at Neuromuscular Synapses

**DOI:** 10.1523/ENEURO.0232-17.2017

**Published:** 2017-08-10

**Authors:** Amanda L. York, James Q. Zheng

**Affiliations:** 1 Department of Cell Biology, Emory University School of Medicine, Atlanta, GA 30322; 2 Department of Neurology, Emory University School of Medicine, Atlanta, GA 30322; 3 Center for Neurodegenerative Disease, Emory University School of Medicine, Atlanta, GA 30322

**Keywords:** NMJ, junctional folds, super-resolution microscopy, synaptic receptors, spatial distribution

## Abstract

The neuromuscular junction (NMJ) is a chemical synapse formed between motoneurons and skeletal muscle fibers. The vertebrate NMJ uses acetylcholine (ACh) as the neurotransmitter and features numerous invaginations of the postsynaptic muscle membrane termed junctional folds. ACh receptors (AChRs) are believed to be concentrated on the crest of junctional folds but their spatial organization remains to be fully understood. In this study, we utilized super-resolution microscopy to examine the nanoscale organization of AChRs at NMJ. Using Structured Illumination Microscopy, we found that AChRs appear as stripes within the pretzel-shaped mouse NMJs, which however, do not correlate with the size of the crests of junctional folds. By comparing the localization of AChRs with several pre- and postsynaptic markers of distinct compartments of NMJs, we found that AChRs are not distributed evenly across the crest of junctional folds as previously thought. Instead, AChR stripes are more closely aligned with the openings of junctional folds as well as with the presynaptic active zone. Using Stochastic Optical Reconstruction Microscopy (STORM) for increased resolution, we found that each AChR stripe contains an AChR-poor slit at the center that is equivalent to the size of the opening of junctional folds. Together, these findings indicate that AChRs are largely localized to the edges of crests surrounding the opening of folds to align with the presynaptic active zones. Such a nanoscale organization of AChRs potentially enables trans-synaptic alignment for effective synaptic transmission of NMJs.

## Significance Statement

Vertebrate neuromuscular synapses are structurally unique as the postsynaptic muscle membrane forms numerous folds in the sarcolemma that are believed to play an important role in synaptic transmission. Acetylcholine receptors (AChRs) are believed to be concentrated on the crest of junctional folds, but their spatial distribution in relation to the junctional folds remain to be fully understood. In this study, we provide evidence that AChRs are not uniformly distributed across the crest of junctional folds, but instead locally enriched at the edges, aligning with the presynaptic active zone. Such a nanoscale organization positions AChRs for effective reception of neurotransmitters released by presynaptic motor terminals during synaptic transmission.

## Introduction

The coordinated anatomic movement of human’s everyday lives depends on rapid and precise neuromuscular communication. At the heart of this process lies the neuromuscular junction (NMJ), a specialized synapse between a motor neuron and a single muscle fiber. The NMJ is a large and topographically complex synapse compared to synapses of the central nervous system (Sanes and Lichtman, 1999; Wu et al., 2010; Shi et al., 2012). As with many biological systems, the organization and structural integrity of the NMJ is critical to its function. Defects in synaptic architecture, including misalignment of the pre- and postsynaptic terminals, are a common phenotype of many neuromuscular diseases and genetic defects (Hirsch, 2007; Slater, 2008; Ha and Richman, 2015), as such mapping the precise organization of synaptic components is crucial to fully understand NMJ function in healthy individuals.

The vertebrate NMJ is marked by unique structural features. Notably, the axon terminal of a motor neuron sinks into the muscle membrane, creating a characteristic depression referred to as the primary gutter. Within this gutter, are smaller invaginations of the postsynaptic membrane, termed junctional folds. The junctional folds can be further divided into the crest, representing the top of the folds closest to the presynaptic terminal, and the trough, representing the bottom part of the infolded membrane. While the exact function of the junctional folds remains to be fully determined, they likely play a critical role in neurotransmission by providing a platform for the spatial segregation of key postsynaptic molecules. For instance, acetylcholine receptors (AChRs), are present at the crest and partially down the sides of junctional folds, whereas voltage-gated sodium channels (VGSCs) are concentrated at the trough (Fertuck and Salpeter, 1974; Matthews-Bellinger and Salpeter, 1983; Flucher and Daniels, 1989). Mathematical modeling of NMJ neurotransmission in the presence or absence of junctional folds suggests that they may act to reduce the threshold necessary for action potential firing, thus making NMJ neurotransmission more efficient (Martin, 1994).

The alignment of neurotransmitter release sites on the presynaptic terminal with the clusters of neurotransmitter receptors on the postsynaptic membrane represents an important mechanism that ensures efficient and effective neurotransmission in the synapses of the central nervous system (Tang et al., 2016). In vertebrate NMJs, the neurotransmitter ACh is released from highly specialized sites on the presynaptic terminal called active zones (Nishimune, 2012). Readily releasable ACh vesicles are docked at the active zones by interacting with the macromolecules in the active zone material (Harlow et al., 2013). Intriguingly, electron microscopy (EM) studies have shown that presynaptic active zones are positioned apposed to the openings of junctional folds (Couteaux and Pecot-Dechavassine, 1970; Dreyer et al., 1973; Patton et al., 2001), whereas AChRs appear to be present across the fold crest and partially down the sides of the infolded membrane (Fertuck and Salpeter, 1974; Matthews-Bellinger and Salpeter, 1983). If this configuration indeed reflects the physiologic organization of the NMJ, it would mean that AChRs are, in large part, misaligned from presynaptic active zones, a finding that would theoretically reduce neurotransmission efficiency. While these EM studies provided many seminal insights into the general organization of NMJ components, a detailed analysis of AChR distribution along the junctional folds has previously been precluded by the relatively sparse labeling that occurs with immuno-EM, and the limited resolution of conventional light microscopy.

In this study, we used super-resolution microscopy to examine the nanoscale distribution of AChRs on the postsynaptic membrane. Together with specific markers for distinct compartments of the NMJ, we present evidence that AChRs are not evenly distributed across the crest of the junctional folds, as the previous model predicts. Rather, our data reveal that AChRs are concentrated at the edge of the crest, apposed to the active zone of presynaptic terminals. As a result, the individual AChR-rich stripes seen using conventional fluorescence microscopy are actually composed of AChRs from the edges of two adjacent crests, whereas the AChR-poor space actually represents the central region of the crest, rather than the region between neighboring crests. Collectively, the results from our data builds a new model whereby AChRs on the postsynaptic membrane are concentrated under presynaptic neurotransmitter release sites, allowing for effective synaptic transmission.

## Materials and Methods

### Antibodies and chemical reagents

The following antibodies were used in this study: rabbit anti-α tubulin (1:200; Abcam, ab15246), mouse anti-rapsyn (1:100; EMD Millipore, MAB2238), rat anti-integrin α7 (1:200; R&D Systems, MAB3518), mouse anti-sodium channel (1:100; Sigma, S8809), rabbit anti-piccolo (1:500; Synaptic Systems, 142 003). Alexa Fluor 488-conjugated α-bungarotoxin (α-BTX) and Alexa Fluor 647-conjugated α-BTX (1:1000) were purchased from Invitrogen (B13422 and B35450, respectively).

### Whole-mount immunofluorescence

Wild-type C57BL/6 mice of mixed sex were sacrificed by exposure to CO_2_. The pretzel-shaped distribution of AChRs as well as the junctional folds are fully developed by postnatal day 21 (Slater, 1982; Singhal and Martin, 2011). Therefore, mice between four and eight weeks of age were used for all experiments. All procedures were conducted in accordance with National Institutes of Health guidelines for animal use and were approved by the Institutional Animal Care and Use Committee of Emory University. The transversus abdominis (TVA) muscle was dissected as previously described by Murray et al. (2014). Briefly, the entire abdominal musculature was dissected from the mouse and immediately fixed for 10 min in 4% (v/v) paraformaldehyde (Polysciences) in PBS. Following fixation, the superficial layers of muscle were removed revealing the TVA muscle situated in the deepest layer of the abdominal wall. The TVA muscle is a very thin muscle group, making it a great candidate for successful whole-mount immunostaining. After the TVA was carefully cleaned of any fat or fascia, the tissue was incubated with Alexa Fluor 488- or Alexa Fluor 647-conjugated α-BTX for 30 min to label AChRs. The tissue was then permeabilized with 2% Triton X-100 (Sigma) for 30 min, and blocked with 4% bovine serum albumin and 1% Triton X-100 in PBS for at least 30 min. The tissue was then incubated with primary antibodies diluted in blocking buffer overnight at 4°C. Following extensive washing with PBS, the tissue was incubated with secondary antibodies in blocking buffer for 2-4 h. Following extensive washing with PBS, the tissue was directly mounted onto a glass slide with Fluoromount-G (SouthernBiotech). All NMJs were imaged “en face.”

For stochastic optical reconstruction microscopy (STORM) imaging, teased muscles were labeled with Alexa Fluor 647-conjugated α-BTX and imaged in a photoswitchable imaging buffer containing cysteamine (MEA), glucose, glucose oxidase, and catalase, all obtained from Sigma-Aldrich (Dempsey et al., 2011). The teased muscle fibers were weighed down on a homemade glass bottom dish to allow for better imaging of en face NMJs.

### Microscopy and image analysis

Laser-scanning confocal images were collected on a Nikon C1 confocal system based on the Nikon Eclipse TE300 inverted microscope (Nikon Instruments) equipped with a 60×/1.4 numerical aperture (NA) Plan Apo oil immersion objective. 3D-structured illumination microscopy (3D-SIM) was performed on a Nikon N-SIM Eclipse Ti-E microscope system equipped with Perfect Focus, 100×/1.49 NA oil immersion objective, and an EMCCD camera (DU-897, Andor Technology). STORM was performed using a Nikon Ti-E total internal reflection fluorescence (TIRF) inverted microscope equipped with Perfect Focus, 488- and 647-nm lasers, and an iXon 897 EMCCD camera (Andor). Images were acquired using a 100×/1.45 N.A. Plan Apo λ objective. Approximately 40,000 frames were collected using TIRF excitation. Images were reconstructed in Nikon Elements.

Intensity profile line scans were performed using the original data in Nikon Elements software. The lines were drawn perpendicular to AChR stripes, near the midline of the primary gutter. For piccolo staining specifically, care was taken to place lines in an area where piccolo puncta are present. A 12-pixel line width was used to average intensities at each point along the line to reduce signal noise. From the intensity profile line scans, the full width at half maximum of individual AChR stripes was used to quantify the width of AChR stripes. The distance between AChR stripes (i.e. the width of fluorescently poor space between AChR stripes) was quantified using profile line scans as follows: 1) measuring the distance between the centers of two neighboring AChR stripes, and 2) subtracting the average width of AChR stripes from the center-to-center distance. Maximum intensity z-projections were created using ImageJ software (National Institutes of Health). Colocalization analysis was performed on Imaris 8.4 software (Andor) using maximum intensity z-projected images. A mask of the synaptic area was created to selectively analyze the degree of colocalization within the synaptic area.

#### Transmission EM

Mouse TVA muscles were fixed with 2.5% glutaraldehyde in 0.1M cacodylate buffer (pH 7.4). Samples were then washed and postfixed with 1% osmium tetroxide in the same buffer for 1 h. After rinsing with deionized water, samples were dehydrated through an ethanol series and then placed in 100% ethanol. Following dehydration, muscle samples were infiltrated with 100% ethanol and Eponate 12 resin (Ted Pella) at a 1:1 ratio overnight. After additional infiltration in Epnonate 12 resin, muscle samples were placed in labeled Beem capsule and polymerized in a 60°C oven. Ultrathin sections were cut at 70-80 nm thick on a Leica UltraCut S ultramicrotome (Leica Microsystems). Grids with ultrathin sections were stained with 5% uranyl acetate and 2% lead citrate. Ultrathin sections were imaged on a JEOL JEM-1400 transmission EM (JEOL) equipped with a Gatan US1000 CCD camera (Gatan).

### Experimental design and statistic analysis

All the data were collected from at least three replica of independently prepared samples. Quantified data were statistically analyzed using one-way ANOVA. The data follow a normal distribution as examined by Anderson-Darling test; *p* values are provided in the corresponding figure legends.

## Results

In this study, we sought to use super-resolution fluorescence microscopy techniques to analyze the spatial distribution of AChRs at the NMJ. The anatomy of muscle tissue can present many hurdles in achieving clean and successful whole-mount immunostaining. While AChRs can be readily labeled by fluorescently tagged α-BTX, immunolabeling of intracellular molecules is complicated by the thick fascia that encapsulates muscle fibers. This thick fascia can limit antibody penetration and generate a significant level of background fluorescence. Here, we used the TVA muscle, a thin and flat muscle located within the abdominal musculature, and adapted a whole-mount protocol (Murray et al., 2014) with modifications to improve antibody penetration and immunolabeling of molecules at NMJs (Fig. 1*A*). Consistently, AChRs are seen concentrated at the motor neuron endplate, appearing as “pretzels” in the center area of the muscle fibers when imaged en face by laser scanning microscopy (Fig. 1*B*). An increase in magnification shows that AChRs are not distributed uniformly within the pretzel pattern of NMJ but the particular spatial organization is not well resolved using conventional light microscopy. With the modified protocol, we were able to label the microtubule network in the intact muscle fibers (Fig. 1*B*). Consistent with previously published results, microtubules form a cage-like network surrounding the postsynaptic area (Schmidt et al., 2012). Therefore, this TVA muscle preparation and modified staining protocol allow effective antibody penetration for immunolabeling of intracellular proteins in conjunction with surface AChR labeling.

**Figure:**
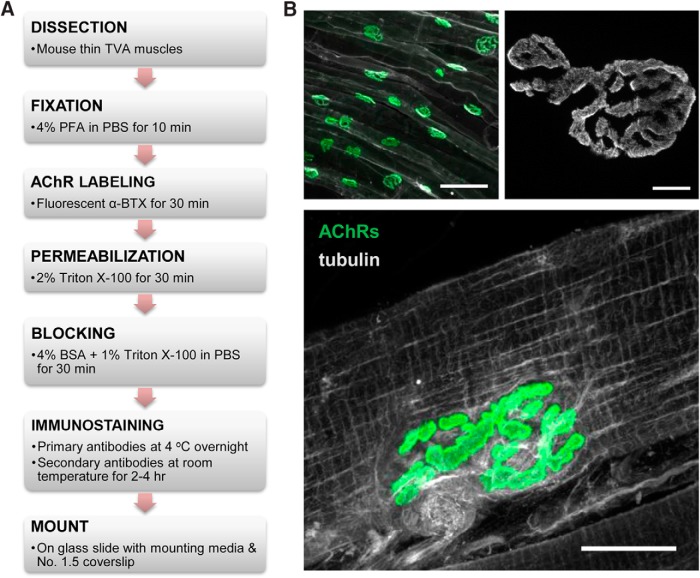
Figure 1. Whole-mount immunostaining of the TVA muscle for reliable detection of antigens at the NMJ. ***A***, The flowchart depicting the protocol used for clean and reliable immunostaining of muscle fibers. ***B***, Example images of the immunostaining. Top left, A low magnification image shows NMJ innervation patterns along the TVA muscles. Integrin α7 (white), used to highlight the membrane of individual muscle fibers, was colabeled with AChRs (green) to highlight the innervation pattern along the TVA muscle. Scale bar, 100 µm. Top right, A high magnification image of an individual NMJ shows the pretzel-shaped AChR distribution at the NMJ of a TVA muscle. Note that AChRs are not uniformly distributed. Scale bar, 5 µm. Bottom: AChRs (green) were colabeled with tubulin (white), an intracellular antigen, to show that the whole-mount method enables excellent antibody penetration to label the microtubule network inside the skeletal muscle. Scale bar, 20 µm.

**Figure 2.**
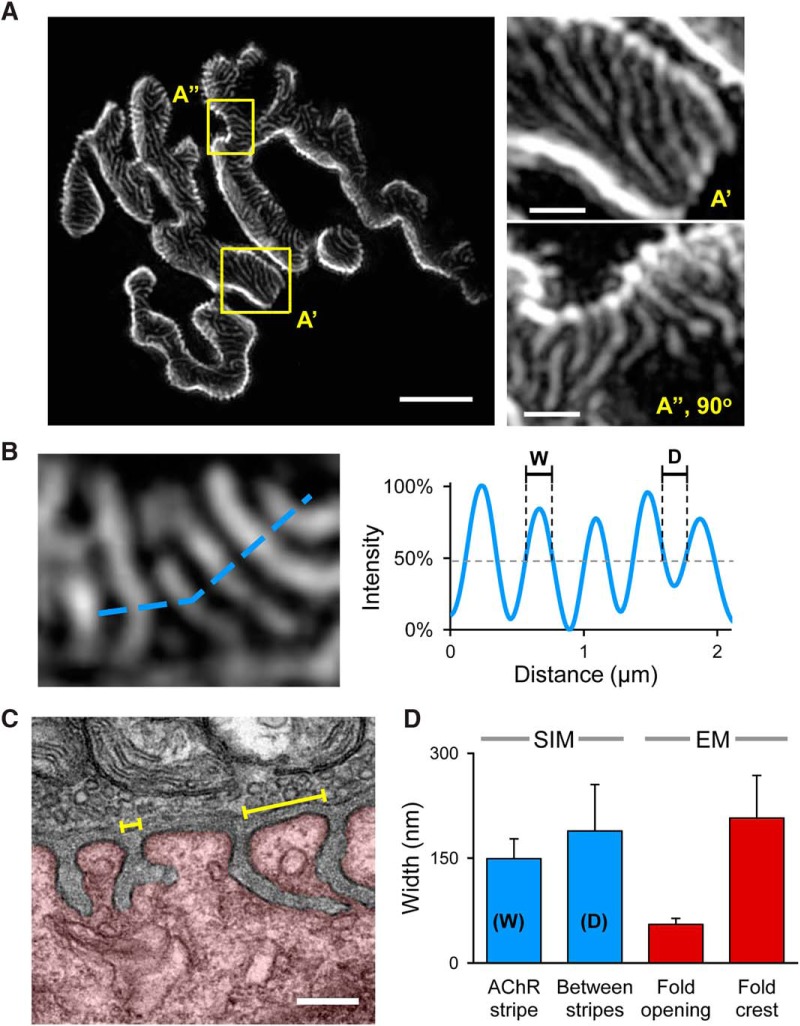
AChRs are distributed in stripes that are not correlated with the crest of NMJ junctional folds. ***A***, A representative 3D-SIM image showing the AChR-rich stripes separated by dark bands. Scale bar, 5 µm. The areas enclosed by yellow rectangles (***A’***, ***A”***) are shown in a high magnification on the right. Scale bar, 1 µm. ***B***, A small region of the AChR stripes (left panel) are used to generate the intensity profile shown on the right. The width of the AChR stripes (W) and the distance between two adjacent stripes (D) are measured and presented in the bar graph in ***D***. ***C***, A representative transmission electron micrograph of an NMJ from the TVA muscle. The junctional folds are clearly visible at the postsynaptic compartment (highlighted by red color). Numerous synaptic vesicles and mitochondria are present within the opposing presynaptic terminal. The average width of junctional fold openings and fold crests (yellow brackets) were manually quantified and presented in the bar graph in ***D***. Scale bar, 0.2 µm. ***D***, The bar graph summarizing the measurement results from the SIM data (blue bars, *n* = 4, >180 stripes) and EM data (red bars; *n* = 7, >40 folds). Error bars represent the SD.

To better resolve the AChR distribution inside the synaptic gutters, we used 3D-SIM, which increases the resolution limit by approximately two-fold compared to conventional light microscopy (Gustafsson, 2000; Gustafsson et al., 2008). Using SIM imaging we found that AChRs are distributed in a “stripe” pattern, where highly fluorescent AChR stripes are separated by fluorescently poor space (appearing as dark bands; Fig. 2*A*). Since AChRs are thought to be concentrated across the crests of junctional folds and absent from the trough of junctional folds, the highly fluorescent AChR stripes could represent the crests of junctional folds separated by the AChR-poor infolded region. To examine this possibility, we performed quantitative analysis on the widths of the AChR stripes and the dark bands by generating intensity profiles (Fig. 2*B*). Our data show that the average width of the AChR stripes is 149 ± 28 nm (mean ± SD), whereas the AChR-poor bands have a width of 188 ± 66 nm. To determine if these numbers represent the crests or the openings of junctional folds, we performed transmitted EM (TEM) on TVA muscles and quantified the average widths of the junctional fold crests and openings (Fig. 2*C*). We found that the openings of the infoldings have a size of 55 ± 9 nm, which is significantly smaller than the width of the dark bands observed with fluorescent staining (Fig. 2*D*). Even considering the difference in resolution limits, the width of the fluorescently-poor dark bands between stripes is significantly larger and does not correlate with the opening of the membrane infoldings. However, the average distance between AChR stripes (188 ± 66 nm) correlates with the average width of fold crests from EM data (207 ± 61 nm) within the margin of error (Fig. 2*D*). Therefore, the AChR-rich and AChR-poor stripes are not directly related to the crests and openings to troughs, respectively.

**Figure 3.**
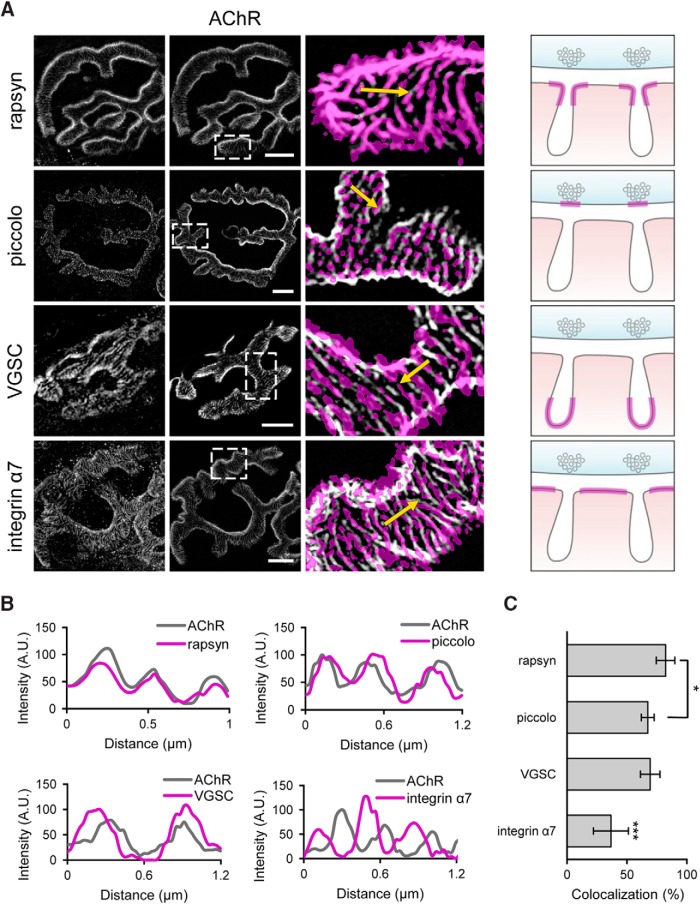
Correlation of AChR distribution with specific pre- and postsynaptic markers at NMJs. ***A***, ***B***, Colocalization of AChRs (white) with various synaptic markers (magenta, threshold). Representative fluorescent images are shown on the right and the schematics on the right depict the known localization of each synaptic marker (magenta line) with respect to the junctional folds. Scale bars, 5 µm. The representative intensity profiles of AChRs (gray) and each of the synaptic markers (magenta) are shown in ***B***. Four markers were examined: Rapsyn, an intracellular AChR scaffolding protein; piccolo, an active zone component; integrin α7, an integrin subunit involved in adhesion in NMJs. ***C***, Quantification of the colocalization of each marker with AChRs. One-way ANOVA analysis: *p* = 2.22 × 10^−5^ (*n* = 4). Error bars represent the SD. Bonferroni analysis: *p = 0.012, ***p < 0.004.

To better understand the relationship between the AChR stripes and junctional folds, we sought to compare the subsynaptic distribution of AChRs to various markers with known localizations. We first compared the subsynaptic localization of AChR and rapsyn. Rapsyn is a highly characterized intracellular protein that immobilizes AChRs at the postsynaptic membrane through scaffolding connections with the underlying actin network (Walker et al., 1984; Antolik et al., 2007). Therefore, we would expect rapsyn to exhibit a very high degree of colocalization with AChRs. Indeed, immunofluorescence of AChRs and rapsyn revealed that they precisely overlap with one another (Fig. 3*A*, top row). Intensity line profiles of these two signals greatly overlap, supporting the visual impression of colocalization (Fig. 3*B*), which is further confirmed by the colocalization analysis (Fig. 3*C*, Manders coefficient = 82.2 ± 7.7%).

Previous EM studies have shown that active zones on the presynaptic terminal precisely align with the opening of the postsynaptic infoldings (Couteaux and Pecot-Dechavassine, 1970; Dreyer et al., 1973; Patton et al., 2001). Therefore, we used an antibody specific for the active zone protein piccolo to mark the area representing the opening of postsynaptic membrane infoldings. Previous studies have shown that immunofluorescence of active zones at mammalian NMJs appear as discrete puncta (Chen et al., 2012). Consistent with the literature, our immunostaining for piccolo revealed a punctate pattern marking the active zones of the mammalian NMJ. When analyzing the piccolo and AChR staining together, we found that the vast majority of piccolo puncta are localized on top of AChR stripes (Fig. 3*A*, second row). A representative profile line scan depicts the overlapping nature of piccolo and AChR staining (Fig. 3*B*). Additionally, colocalization analysis reveals piccolo overlaps with AChRs to a high degree (Manders coefficient = 67.3 ± 5.4%; Fig. 3*C*). These data suggest that AChR stripes may be localized to the area surrounding the infolded membrane region. We also compared the localization of AChRs relative to the trough of junctional folds. Here, we double-labeled AChRs and VGSCs, which have been shown to be spatially restricted to the trough of junctional folds (Flucher and Daniels, 1989). VGSC staining was found to largely overlap with AChR stripes (Manders coefficient = 69.4 ± 8.1%; Fig. 3*A*,*C*), which is further confirmed by the profile line scan (Fig. 3*B*). These data, in conjunction with piccolo data, supports the hypothesis that AChRs may be spatially restricted to the area immediately surrounding the opening of infolded membrane.

To further test this hypothesis, we compared the localization of AChRs relative to the junctional fold crests. Integrin α7 is a muscle specific transmembrane receptor that links the cell to the surrounding extracellular matrix (Song et al., 1992; Song et al., 1993). In the mature NMJ, integrin α7 is restricted to the crest of junctional folds (Martin et al., 1996; Schwander et al., 2004). Therefore, we labeled integrin α7 to mark the junctional fold crest and compared this staining to that of AChRs. Similar to AChRs, integrin α7 also exhibits a similar stripe staining pattern (Fig. 3*A*). However, when overlaid with AChR staining it is apparent that integrin α7 and AChRs occupy distinct domains from one another. Profile line scans depict the alternating pattern of AChR and integrin α7 staining, where integrin α7 is primarily present in the space between AChR stripes (Fig. 3*B*). Additionally, integrin α7 was found to colocalize with AChRs to a much lower degree (Manders coefficient = 36.6 ± 14.6%) than that of piccolo and VSVGs (Fig. 3*C*), suggesting that integrin and AChRs are distributed into distinct nanoscale domains. It should be noted that teased muscle fibers did not have the exactly same orientation for en face imaging of NMJs. As a result, a small tilt in the angle of the muscle fiber orientation could affect colocalization analysis due to the 3D nature of the sample. We believe that this is one of the contributing factors to the “imperfect” colocalization results observed here. Nevertheless, these findings suggest that AChRs and integrin α7 occupy different domains within the junctional folds. Taken together, these data suggest that the localization of AChRs may be restricted to the area immediately surrounding the infolded region and thus absent from the center most part of the fold crest.

**Figure 4.**
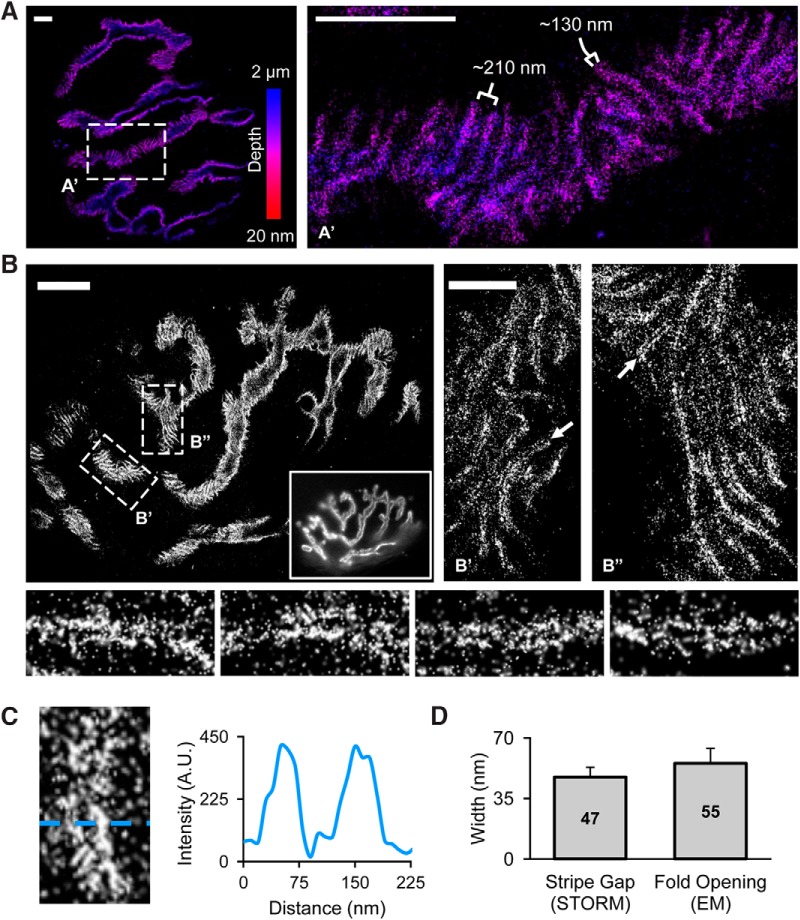
Super-resolution imaging reveals AChRs are concentrated around the opening of junctional folds. ***A***, 3D-STORM imaging of AChRs highlights the 3D nature of the postsynaptic membrane. The widths of AChR stripes (∼130 nm) and the distance between stripes (∼210 nm) is consistent with quantifications from our previous SIM data. A small region outlined by dashed rectangle (***A’***) is shown in a higher magnification on the right. Color scale bar indicates Z-depth. Scale bars, 2 µm. ***B***, Representative STORM images of AChRs at a NMJ. Scale bar, 5 µm. Inset image shows the same NMJ imaged using widefield microscopy. Close-up regions (***B’***, ***B”***) reveals a thin slit at the center of each AChR stripe (arrow). Scale bar, 1 µm. Bottom, close-up view of individual AChR stripes. ***C***, Representative profile linescan further highlighting the slit at the center of each AChR stripe. ***D***, Quantification of the width of the AChR stripe gap (measured from STORM data, *n* = 23) and the width of the junctional fold opening (measured from our TEM data, *n* = 50). Error bars represent the SD. Average width values at center of each bar.

Based on previous studies and our results thus far, we hypothesize that AChRs are concentrated at the edge of junctional fold crests and part way down the sides of the infolded membrane. Since the opening of infoldings is below the resolution limit of conventional fluorescence microscopy and SIM, the fluorescent signals from two edges of neighboring crests may combine to give the appearance of a single AChR stripe. To test our hypothesis, we used STORM, which has a theoretical resolution limit of ∼10-20 nm in the *xy*-axis and 50 nm in the *z*-axis (Rust et al., 2006), providing us with the resolution necessary to visualize the opening of junctional folds. Similar to our SIM data, AChR staining appears as stripes within the overall pretzel pattern of the NMJ (Fig. 4*A*). Additionally, the size and distance between AChR stripes is consistent with our previous SIM data at ∼130 and 210 nm, respectively. Upon closer analysis of the AChR stripes, we observed a thin, fluorescently poor slit that runs down the center of each stripe (Fig. 4*B*, arrows). This slit at the center of AChR stripes is clearly highlighted in a profile line scan of a representative stripe showing the decrease in AChR fluorescence at the center of the AChR stripe (Fig. 4*C*). On average, the width of the slit is 47 ± 5.7 nm (Fig. 4*D*), which is approximately equivalent to the average width of the openings of infoldings from our EM data (55 ± 8.5 nm). Therefore, the slit running down the center of each AChR stripe likely represents the opening of the infolding of the postsynaptic membrane. This is the first evidence visualizing the opening of membrane infoldings using fluorescence microscopy. Overall, these data support the hypothesis that AChRs are concentrated at the edge of the junctional fold crests, surrounding the opening of membrane infoldings.

## Discussion

**Figure 5.**
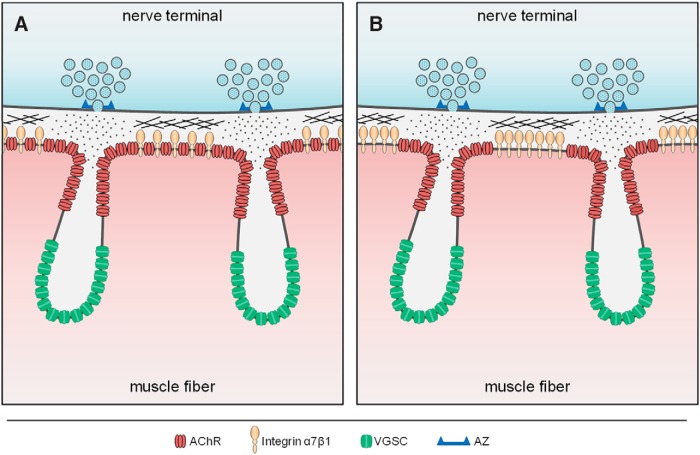
Schematics showing the current view (***A***) and the proposed revision (***B***) of AChR distribution along junctional fold crests. Classically, it is believed that AChRs (red) are distributed across the entire junctional fold crest and partially down the sides of the infolded membrane and excluded from the trough of junctional folds where VGSC (green) are localized (***A***). However, we propose that AChRs are instead spatially restricted to the area immediately surrounding the opening of junctional folds and are segregated from the adhesion molecule integrin α7β1 (tan) located at the center-most part of fold crests (***B***). The spatial segregation of AChRs and integrin α7β1 could be beneficial to maintaining strong synaptic adhesion between the pre- and postsynaptic terminals. Furthermore, this subsynaptic organization would position AChRs directly opposite that of the active zone (AZ, blue bracket), and thus, in the prime position to receive and respond to acetylcholine (dots) release.

AChRs are highly concentrated on the postsynaptic membrane of the NMJ for effective neurotransmission during muscle contraction. The large NMJs in vertebrates are structurally unique, as the postsynaptic muscle membrane forms numerous folds in the sarcolemma that are believed to play an important role in synaptic transmission (Martin, 1994; Shi et al., 2012). Not only do the junctional folds effectively increase the postsynaptic surface area, but they also provide a platform for the spatial segregation of molecules involved in distinct signaling pathways of NMJ neurotransmission. For example, AChRs are concentrated at the fold crest, whereas VGSCs are localized to the troughs of junctional folds (Flucher and Daniels, 1989). The topography of the folds and the spatial segregation of key postsynaptic proteins within them act to facilitate the amplification of synaptic current (Martin, 1994), thus allowing for more efficient neurotransmission. Previous studies using EM and light microscopy have shown that AChRs are distributed along the crest and part way down the junctional folds, but excluded from the trough (Fertuck and Salpeter, 1974; Burden et al., 1979; Matthews-Bellinger and Salpeter, 1983; Flucher and Daniels, 1989; Marques et al., 2000). While such a distribution of AChRs would position them close to the presynaptic membrane, it would not provide the best configuration for synaptic transmission given that the presynaptic active zones are not aligned with the center of the crest, but rather with the opening of membrane infoldings (Couteaux and Pecot-Dechavassine, 1970; Dreyer et al., 1973). Unfortunately, conventional light microscopy lacks the resolution to resolve nanoscale details of AChR distribution. While EM has the nanoscale resolution, immunogold labeling tends to be sparse and biochemical reaction products of horseradish peroxidase may not be spatially confined. In this study, we present evidence, using two different types of super-resolution fluorescence imaging, that AChRs are concentrated at the edges of the crests of junctional folds. In this case, fluorescence from the two edges of adjacent crests comprises a single AChR stripe observed by conventional light microscopy, whereas the fluorescently poor space (the dark band) between AChR stripes actually represents the top of the fold crest. It should be noted that our data are presented as maximum intensity projections, thus the results are unlikely affected by the summation of signals on the wall of the folds. However, the *z*-axis resolution is known to be limited, thus we cannot rule out the possibility that en face imaging might not fully resolve AChR distribution on the wall of junctional folds. Nonetheless, the concentration of AChRs at the edges of the crests essentially creates a trans-synaptic alignment of AChRs with the presynaptic active zones where the neurotransmitter ACh is released (Fig. 5). Concentrating AChRs at the crest edge would likely provide more efficient delivery of ACh to AChRs, and thus more efficient neurotransmission, rather than evenly distributing AChRs across the fold crest. A similar trans-synaptic alignment of active zones and neurotransmitter receptors has also been reported in synapses within the central nervous system (Tang et al., 2016), suggesting this trans-synaptic alignment may represent a conserved mechanism for efficient neurotransmission across various synapses.

Strong synaptic adhesion is important for the integrity of the synapse. One of the key synaptic adhesion molecules at the NMJ is integrin α7, which associates with integrin β1 to form integrin α7β1 (Mayer, 2003; Singhal and Martin, 2011). Integrin α7β1 binding of laminin α4 at NMJs is crucial for the positioning of active zones, such that the loss of laminin α4 results in the misalignment of active zones with junctional folds (Patton et al., 2001; Sanes, 2003). Our data show that integrin α7 and AChRs appear to occupy largely discrete domains, although both are localized at the crest of junctional folds. The localization of integrin α7β1 to the center most part of the fold crest could represent a specialized area for synaptic adhesion. It is possible that localizing integrin molecules into distinct nanodomains could enable stronger synaptic adhesion because adhesion molecules would not be interspersed with various other postsynaptic molecules. Additionally, a dense patch of adhesion molecules interacting with the presynaptic terminal through binding to the synaptic basal lamina could also act as a “barrier,” restricting the diffusion of ACh to the area of fold openings where AChRs are concentrated. Clearly, future research is needed to evaluate these possibilities.

The exact function of the junctional folds remains unclear. Many studies have found mutations that result in the loss of junctional folds (Noakes et al., 1995; Barik et al., 2014). However, these mutations usually result in disruptions to various other parts of the postsynaptic compartment, including AChR expression, making the study of junctional fold function very difficult. Nonetheless, studies using mathematical modeling of the NMJ have provided insight into the function of junctional folds. Modeling of neurotransmission with and without junctional folds has suggested that the folds act to amplify synaptic current following ACh release (Martin, 1994). Specifically, the amount of ACh quanta required to initiate muscle contraction is approximately double in the absence of junctional folds (Martin, 1994). Thus, in the presence of junctional folds, fewer AChRs need to be activated to initiate muscle contraction. Furthermore, densely clustering AChRs directly opposite that of neurotransmitter release sites could provide another mechanism to further increase the efficacy of NMJ neurotransmission. Currently, there are no experimental manipulations available that can alter AChR distribution without affecting NMJ structure. However, it is exciting to speculate that tools may be developed in the future allowing detailed analysis of neurotransmission with AChRs concentrated at the edge of the infoldings versus AChRs distributed across the fold crest.

Defects in junctional folds are a common phenotype of many diseases affecting the NMJ (Hirsch, 2007; Ha and Richman, 2015). For example, patients with myasthenia gravis produce antibodies targeting key postsynaptic molecules, of which AChRs are most often targeted (Hirsch, 2007; Spillane et al., 2010; Ha and Richman, 2015). Binding of the autoimmune antibodies results in the internalization of AChRs, reducing their concentration to about one-third that of normal NMJs (Lindstrom, 2000). AChR–antibody binding also results in damage to the postsynaptic membrane, which typically has reduced folds and a widened synaptic cleft (Hirsch, 2007). Consequently, patients exhibit severe muscle weakness due to a significant disruption in neurotransmission. Therefore, proper muscle function appears to be intricately linked to the structure of the postsynaptic terminal and the localization of AChRs.

In summary, our super-resolution imaging has revealed a distinct nanoscale pattern of AChRs on the postsynaptic membrane of NMJs. A similar nanoscale organization has also been shown in synapses of the central nervous system (Tang et al., 2016), suggesting that nanoscale organization and alignment of presynaptic and postsynaptic components may represent a conserved mechanism to ensure effective synaptic transmission.

*Note Added in Proof:* Some of the funding sources for this article were incorrectly listed in the online Footnotes and on the cover page of the PDF article file. The funding sources in the HTML and PDF versions of the article have now been corrected.

## Synthesis

Reviewing Editor: Darcy Kelley, Columbia University Decisions are customarily a result of the Reviewing Editor and the peer reviewers coming together and discussing their recommendations until a consensus is reached. When revisions are invited, a fact-based synthesis statement explaining their decision and outlining what is needed to prepare a revision will be listed below.

This manuscript was transferred from JNeurosci. The authors have revised the paper to meet comments from the reviewers and have requested that it be re-reviewed by the same people.

However, the major comment of the reviewers, who agree that the paper is meritorious, is that its significance is not clear. Why does it matter that the AChRs sit at the edges of the tops of the crests of the NMJ folds as opposed to being spread uniformly across the top of the crests? Buried in the paper are two key points: one is that the presynaptic active zones don't match this uniform-across-the-crests distribution and the other is that misalignment of synaptic elements is a key feature of neuromuscular disorders. The paper needs some rewiring and perhaps even a new title, something along the lines of: Super-resolution microscopy revises our view of synaptic alignment at motor synapses'.

The introduction should be re-organized to address:

1) Why it is important to understand the NMJ

2) The conventional view of how the pre- and post-synaptic elements align

3) What this view is based on

4) The problem: are pre- and post-synaptic element really misaligned?

5) Why important?  Neuromuscular diseases affect the synaptic organization.  Need to know what is normal.

This is a very interesting paper but needs focusing. It might be helpful to get some suggestions from colleagues or a science writing consultant.
